# Plasmon Resonance in Photoabsorption of Colloidal Highly Doped ZnO Nanocrystals

**DOI:** 10.1186/s11671-018-2710-3

**Published:** 2018-09-24

**Authors:** Andrey N. Ipatov, Leonid G. Gerchikov, Claude Guet

**Affiliations:** 1 St. Petersburg National Research Academic University RAS, Khlopin Str. 8/3, St., Petersburg, 194021 Russia; 2 Energy Research Institute at Nanyang Technological University (ERI@N) and School of Materials Science and Engineering, Nanyang Technological University, Nanyang Avenue 50, Singapore, 639798 Singapore

**Keywords:** Photoabsorbtion, Plasmon, Nanocrystal

## Abstract

A new type of dipole plasmon excitations in colloidal highly doped ZnO nanocrystals has been studied by means of many-body quantum mechanical approach. We demonstrate that in photodoped ZnO nanocrystals, the conduction band electrons are localized close to the surface and the plasmon oscillations are induced by their angular motion. The transition of this plasmon mode from classical to quantum regime is defined by the nanocrystal size. The size dependence of the resonance frequency which results from quantum effects is in remarkable agreement with experimental observations.

## Background

To a large extent, optical properties of nanoparticles are determined by the presence of localized surface plasmon resonances (LSPR) in their excitation spectra [1–8]. Kriegel et al. [2] published a quite detailed overview of emerging colloidal nanocrystals (NCs), including impurity-doped metal oxide NCs, copper chalcogenide NCs, and degenerately doped semiconductor NCs, and extensively discussed their optical properties as well as applications for sensing, near-field enhancing spectroscopy, tunable optoelectronic devices, or biomedical applications. All these new materials are an alternative to noble metal NCs which have been extensively studied over the last decades; for a comprehensive review, see also Ref. [9]. It has been shown that it is possible to tune the optical response of heavily doped semiconductor nanocrystals (NCs) down to the IR range [10–15], by acting on various control parameters [11, 13, 16–20], thus opening new perspectives for nanophotonics. The main advantage of semiconductor NCs is that the carrier density can be tuned over a wide interval. Whereas a metallic conductor has a fixed electron density, a semiconductor can be doped to achieve arbitrary carrier densities in a range of 10^16^÷10^22^cm^−3^ [10, 13, 21, 22]. Carrier doping allows access to tunable LSPR in a wide frequency range from the THz to IR and visible region [13]. Such a carrier density tunability is a unique property of semiconductor nanoparticles and cannot be achieved using metallic droplets [23–25]. Doping can be done by incorporating various types of impurities into the crystal lattice [7, 10, 18], and the plasmon resonance frequency can be tuned or switched by active control of carriers [13, 16–18, 21, 22]. Moreover, the plasmon frequency and its line shape depends not only on the NC’s carrier density but on the type of doping, which can be “bulk-like” or “surface-like” [15, 22]. In the former case, the charge of free carriers is neutralized by the charge of doping impurities all over the NC volume, while in the latter case, the free carriers are injected into the NC volume by donors/acceptors from the surrounding media located at the NC interface.

Theoretical studies of the optical response of semiconductor nanoparticles have revealed significant differences between quantum mechanical and classical descriptions [5, 22, 24, 26]. As the nanoparticle size decreases, the plasmon resonance shifts to higher energies with visible deviation from classical predictions [5, 21, 22]. Moreover, the dynamic properties of highly doped NCs undergo a transition from the size-quantization regime to the classical regime of plasmon oscillations [22]. It can be observed by varying either the number of carriers or the NC size.

In this work, we present a theoretical analysis of the optical response of photodoped ZnO NCs such as experimentally investigated in Ref. [21, 27]. In these experimental studies, the photoabsorption cross sections of colloidal ZnO NC of fixed sizes and carrier densities were measured. The conduction band photoelectrons were generated in ZnO NC by photodoping process, while the holes were trapped by the hole scavenging centers in surrounding toluene. The mean conducting electron concentration *n*_*e*_ in almost spherical photodoped nanocrystals of different radii (from 1.75 to 6 nm) was found to reach an upper limit of (1.4±0.4)×10^20^cm^−3^ [21, 27]. The intraband absorbance was measured in the range of 0.2 to 1.0 eV, and significant differences with the classical Drude model prediction were observed. The authors showed that a quantum mechanical approach based on single-particle transitions yielded the experimental size dependence of the resonance frequency only qualitatively [21].

The aim of this article is to revisit the theoretical approach of plasmon resonances in photodoped ZnO NCs by going beyond the non-interacting single-particle approximation [21, 27]. It is based on the self-consistent many-body quantum mechanical treatment of conduction electrons within the random phase approximation (RPA) with local exchange [28]. It was shown that free charge carriers in semiconductor nanoparticles form atomic-like shells [29, 30]. We solve the local density approximation (LDA) Kohn-Sham equations to describe the electron shell structure. The electron correlations responsible for the collective plasmon excitations are taken into account within the RPA. We show that the plasmon resonance in ZnO NC differs substantially from the well known Mie resonance in metallic droplets. At variance with bulk-like doped NCs, there is no restoring positive charge in surface-like doped ZnO NC. Consequently, the Coulomb repulsion between free electrons push them close to the NC surface. In turn, this specific electronic configuration leads to a dipole mode, where only the angular degrees of freedom are excited, while the electronic radial motion is not involved. Contrary to the ordinary Mie surface dipole plasmon where electrons undergo pure translational oscillations, electrons in highly doped ZnO NCs undergo tangential vibrations within the rather thin electronic shell in a manner similar to the plasmon oscillations in fullerene molecules [31]. We also show that the transition of this plasmon mode from the classical to quantum confinement regime is governed by the ratio of the NC size to the effective Bohr radius and does not depend on the numbers of free electrons. The quantum effects in plasmon oscillations result in a blue shift of the dipole resonance frequency that nicely agrees with the experimentally observed LSPR size dependence [21].

## Methods

The aim of the study is the theoretical analysis of optical properties of photodoped ZnO nanocrystals. The ground state configurations of the systems with varying numbers of particles were calculated within the local density approximation. The ground state wave functions are single-particle energies which were obtained by self-consistent numerical solving of the set of Kohn-Sham equations [32]. The complete basis of the single-particle states was generated using the the B-spline method [33] by expanding the basis functions in a cavity of large radius over a finite number of B-splines. The cavity radius was chosen equal to the NC radius. The desired accuracy of the computations with the use of the B-spline discrete basis was achieved by the appropriate choice of the number and the order of the B-splines used in the computation. We have used 50 B-splines of the order 7 to achieve sufficient accuracy (10^−5^) of the results. The standard subroutine RG from the eigensystem subroutine package (EISPACK) has been used for the obtaining of the eigenvalues and the eigenvectors of the RPA matrix equation [28], which solution provides us the set of dipole excitation energies and corresponding oscillator strengths. The photoabsorption spectrum has been obtained by the broadening of the calculated oscillation strength distribution by Lorentzian profiles with the fixed folding width.

## Results and Discussion

### Ground State Structure

We consider the system of *N* conduction band electrons localized within the ZnO NC of radius *R*. Following [21], we assume that the number of electrons varies with NC size as *N*=4*πn*_*e*_*R*^3^/3, where the fixed mean electron concentration, *n*_*e*_ = 1.4×10^20^ cm ^−3^, is determined by the highest achievable level in the photodoping process. The radius of the considered NCs ranges from 2.4 to 6 nm; accordingly, the number of conduction electrons, *N*, vary from 8 to 128.

We employ the envelope function approximation to describe the electron motion assuming that *R* is much larger than the lattice constant. It is known that the electronic band structure of bulk ZnO is characterized by a non isotropic and non parabolic energy spectrum [34]. However, for the current problem of collective dynamics of *N* delocalized electrons, we will neglect these small energy spectrum effects and consider an isotropic parabolic energy dispersion with effective mass $m_{e}^{*}=0.3~m_{e}$ [34]. For the same reason, we consider the ZnO NCs as spherical systems.

As the electrons are strongly localized within the ZnO NC volume due to the high conduction band offset at the NC interface [6], we impose all electron wave functions to vanish at the NC border *r*=*R*. Thus, we consider *N* interacting electrons localized within an infinite spherical well which overall charge neutrality is ensured by a positive charge surface distribution that does not create any field inside the NC. The effective Hamiltonian of the considered system is simply:

1 $$ \hat{H} = \sum\limits_{a} \frac{\hat{\mathbf{p}}^{2}_{a}}{2 m_{e}^{*}}+ \frac{1}{2}\sum\limits_{a,b}V\left(\mathbf{r}_{a},\mathbf{r}_{b} \right),   $$

where *V*(**r**_*a*_,**r**_*b*_) stands for the pair electron Coulomb interaction. Its explicit expression, accounting for the polarization of ZnO material and the surrounding media, is written as a multipole expansion [3],

2 $$ \begin{aligned} &V\left(\mathbf{r}_{a},\mathbf{r}_{b} \right) = \sum\limits_{L,M} \frac{4\pi V_{L}}{2L+1} Y_{L M}(\mathbf{n}_{a})Y^{*}_{L M}(\mathbf{n}_{b}), \\ &V_{L} = \frac{e^{2}}{\varepsilon_{i}}\left(\frac{r^{L}_<}{ r^{L+1}_>}+ \frac{\left(\varepsilon_{i}-\varepsilon_{m}\right)\left(L+1\right)\left(r_{a} r_{b}\right)^{L}}{\left(L\varepsilon_{i}+(L+1)\varepsilon_{m}\right) R^{2L+1}} \right), \end{aligned}  $$

where *r*_<_ and *r*_>_ are the smallest and largest of the two radial positions, respectively. ZnO and toluene dielectric constants are assigned their bulk values *ε*_*i*_=3.7 and *ε*_*m*_=2.25 [21], respectively. With these parameters, the effective Bohr radius $a_{0}=\hbar ^{2} \varepsilon _{i}/m_{e}^{*} e^{2} = 0.65$ nm is smaller than the NC radius.

The single-particle electron energies, *ε*_*i*_, and envelope wave functions *ψ*_*i*_ satisfy the set of LDA Kohn-Sham equations,

3 $$ \left[\frac {\hat{\mathbf{p}}^{2}}{2 m_{e}^{*}}+ V_{mf}(\mathbf{r})\right]~ \psi_{i}(\mathbf{r}) = \epsilon_{i} \psi_{i}(\mathbf{r}),   $$

where the mean field potential *V*_*mf*_ is given by the sum of direct, *V*_*D*_(**r**), and exchange, *V*_*x*_(**r**), parts,

4 $$ \begin{aligned} &V_{mf}(\mathbf{r}) = V_{D}(\mathbf{r}) +V_{x}(\mathbf{r}), \\ &V_{D}(\mathbf{r}) = \int V(\mathbf{r},\mathbf{r}^{\prime})\rho(\mathbf{r}^{\prime})d\mathbf{r}^{\prime}, \quad V_{x}(\mathbf{r}) = -\frac{e^{2}}{\varepsilon_{i}}\left(\frac{3\rho(\mathbf{r})}{\pi}\right)^{1/3}, \end{aligned}  $$

with $\rho =\sum _{i} |\psi _{i}|^{2}$ being the electron density. Note that we could replace the local density-dependent exchange term in its Dirac form by a more realistic local density-dependent exchange-correlation term as usually done. We do not do it as the theoretical construction of excited states which will follow automatically accounts for a large part of ground state correlations of RPA nature.

**Fig. 1:**
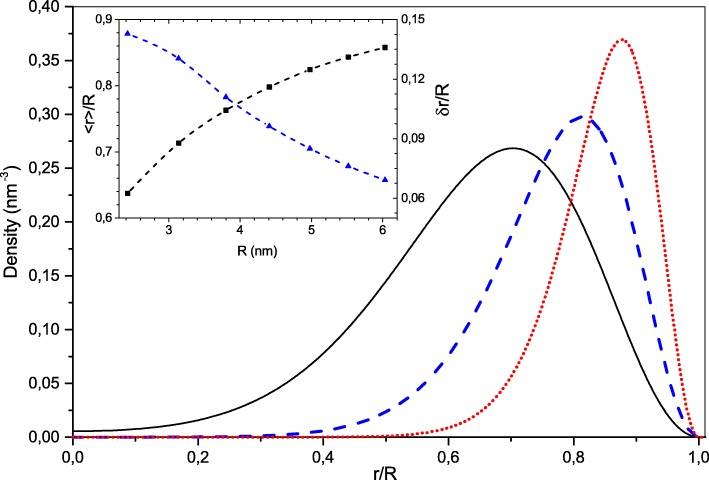
Radial density distributions for NCs with different numbers of delocalized electrons, *N*=18 (solid black like), 50 (dashed blue line), 128 (dotted red line). In the inset, the reduced mean radius (black) and its dispersion (blue) is represented as a function of NC radius

For the sake of simplicity, we consider spherically symmetric electron configurations with closed electronic shells. In this case, the single-particle wave functions are given as the products of radial, angular, and spin components [35]. Consequently, the index *i*=(*n*,*l*), where *n* is the radial quantum number and *l* the angular momentum one. The numerical solution of Eq. (3) for electron number *N*<130 showed that the ground state electron configuration was made of occupied electronic states with lowest radial quantum numbers *n*=1. These electronic states have node-less radial wave functions and differ by the values of angular momenta *l*. Thus, accounting for the spin degeneracy, the “magic” numbers of electrons for such symmetric configurations are *N*=2(*l*_*max*_+1)^2^, where *l*_*max*_ is the maximum angular momentum of the highest occupied electronic state. In Fig. 1, we show the density distributions *ρ*(*r*) for the NC with *N* = 18, 50, 128 electrons. One can see that the radial density distribution becomes more and more narrow and shifts towards the NC interface as the size increases. The insert of Fig. 1 shows the size dependence of the mean electronic value of the electronic radial distribution 〈*r*〉 upon the NC radius *R* and the ratio *δr*/*R*, of its dispersion $\delta r = \sqrt {\langle r^{2} \rangle - {\langle r\rangle }^{2}}$ (that can be considered as the effective electronic shell width) to the NC radius. This ratio which amounts only ∼ 0.15 for the smallest NC with *N*=8 electrons rapidly decreases for the larger systems. Numerically, the width of electronic shell *δr* is about two thirds of the effective Bohr radius *a*_0_ whereas the peak of the electronic shell is shifted from the NC interface due to the quantum reflection by approximately *R*−〈*r*〉≃2*a*_0_. This feature of the electronic system follows from the strong Coulomb repulsion that pushes the electrons to the NC border forming a hollow spherical charge distribution. If NC radius is large enough, the Coulomb repulsion force at the NC border *e*^2^*N*/*ε*_*i*_*R*^2^ becomes much stronger than the centrifugal force $\hbar ^{2}l(l+1)/m_{e}^{*}{\langle r \rangle }^{3}$ even for the highest occupied state with $l=l_{max}=\sqrt {N/2}-1$. One can see that their ratio amounts *a*_0_/2*R*. That is why the radial and angular electron motions separate if *R*≫*a*_0_. In this case, the electronic system is similar to a quantum rotator, e.g., the energy spectrum of occupied single-particle states *ε*_*i*_ of Eq. (3) is well approximated by the formula,

5 $$ \epsilon_{1,l} - \epsilon_{1,0} = \frac{\hbar^{2}l(l+1)}{2m_{e}^{*} {\langle r\rangle}^{2}}.   $$

### Optical Response

In the linear response approximation, an external harmonic electric field induces time-dependent self-consistent fields of same frequency. The knowledge of the corresponding small-amplitude vibrations provides information on the dipole excited states as well as on the transition probabilities between ground state and excited states. For a system whose ground state is a Slater determinant |*Φ*_0_>, the correlated many-body dipole excited states within the RPA approach are constructed as a linear superposition of a particle-hole excitation [36]:

6 $$ |\Phi_{\nu}>=\sum_{i>F,j<F} \left(X_{ij}^{\nu }\hat{a}_{i}^{+}\hat{a}_{j}+Y_{ij}^{\nu }\hat{a}_{j}^{+}\hat{a}_{i}\right) |\Phi_{0}>.  $$

The indices *i*,*m*, (*j*,*n*) refer to the single-particle states above (below) the Fermi level F; $\hat {a}^{+}$ and $\hat {a}$ are the creation and destruction single-particle operators, respectively.

The forward-going, $X_{ij}^{\nu }$, and the backward-going, $Y_{ij}^{\nu }$, amplitudes can be obtained by solving the matrix RPA equation [28]:

7 $$ \left(\begin{array}{ll} \mathbf{A} & \mathbf{B} \\ \mathbf{B}^{\star} & \mathbf{A}^{\star} \end{array} \right) \left(\begin{array}{l} \mathbf{X}^{\nu} \\ \mathbf{Y}^{\nu} \end{array} \right) =\omega_{\nu} \left(\begin{array}{l} \mathbf{X}^{\nu} \\ -\mathbf{Y}^{\nu} \end{array} \right),  $$

where the eigenvalues *ω*_*ν*_ are the excitation energies. The matrixes **A** and **B** are defined as follows

8 $$ {\begin{aligned} A_{ij,mn}&\,=\,\delta_{im}\delta_{jn}\left(\varepsilon_{i}-\varepsilon_{j}\right) \,+\, \left\langle in\left| \hat{v}\right| jm\right\rangle, \quad B_{ij,mn}&\,=\,\left\langle im\left| \hat{v}\right| jn\right\rangle \\ \hat{v}(\mathbf{r},\mathbf{r}^{\prime}) &= V(\mathbf{r},\mathbf{r}^{\prime})+ \delta\left(\mathbf{r}- \mathbf{r}^{\prime}\right)\delta V_{x}/\delta \rho. \end{aligned}}  $$

Note in passing that the backward amplitudes, $Y_{ij}^{\nu }$, measure the contribution of electron-hole ground state correlations $\hat {a}_{j}^{+}\hat {a}_{i}|\Phi _{0}>$, respectively, to the excitation of the excited state |*Φ*_*ν*_> of frequency *ω*_*ν*_.

The corresponding dipole oscillator strengths *f*_*ν*_ are expressed in terms of the RPA amplitudes **X**^*ν*^ and **Y**^*ν*^,

9 $$  f_{\nu} = \frac{2 m_{e}^{*} D^{2}_{\nu}\omega_{\nu}}{\hbar^{2}}, \quad D_{\nu} = \sum_{ij} \left(X^{(\nu)}_{ij} d_{ij} + Y^{(\nu)}_{ij} d_{ji} \right),  $$

where *d*_*ij*_=〈*i*|*z*|*j*〉 are the single-particle dipole matrix elements.

**Fig. 2:**
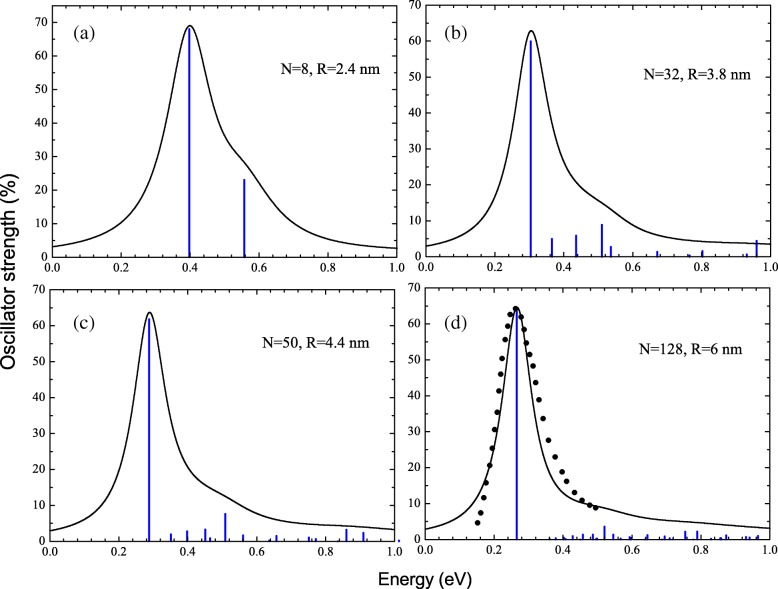
Oscillator strength distributions and corresponding photoabsorbtion peak profiles calculated within RPAE approach for NCs with *N*=8 (**a**), 32 (**b**), 50 (**c**), and 128 (**d**) conduction band electrons. Comparison of experimental [21] (black squares) and calculated (solid line) resonance peak profiles for NC with *R*≈6 nm (**d**)

The photoabsorption cross sections have been obtained by broadening the calculated oscillator strength distribution by Lorentzian profiles with the folding width of 0.2 *ω*. Photoabsorption spectra together with oscillator strength distributions for NCs with *N*=8 (a), 32 (b), 50 (c), and 128 (d) electrons are shown in Fig. 2. In Fig. 2d, we compare the calculated photoabsorption cross section with the experimental data of Ref. [21] for ZnO NC with *R*≃ 6 nm. One can see that in all considered NCs, the spectra are dominated by a single resonance line, which position actually determines the maximum of the photoabsorption cross section. Indeed, it is expected since the single-particle electronic spectrum is similar to a quantum rotator. The strongest transitions happen for a maximal wave function overlapping, i.e., with equal radial quantum number *n*. In our case, there is only one such optical transition from the highest occupied state *j*=(1,*l*_*max*_) to the lowest vacant state *i*=(1,*l*_*max*_+1). However, the corresponding dipole excitation strongly differs from the non-interacting single-particle transition whenever a strong Coulomb repulsion makes electron correlations important and overwhelming. This is the case when *R*≫*a*_0_. Namely, the energy of collective excitation, *ω*_*ν*_, exceeds the energy of single-particle transition

10 $$ \Delta = \left(\epsilon_{1, l_{max}+1} -\epsilon_{1, l_{max}}\right)=\frac{\hbar^{2} (l_{max}+1)}{m_{e}^{*}{\langle r \rangle}^{2}}.   $$

**Fig. 3:**
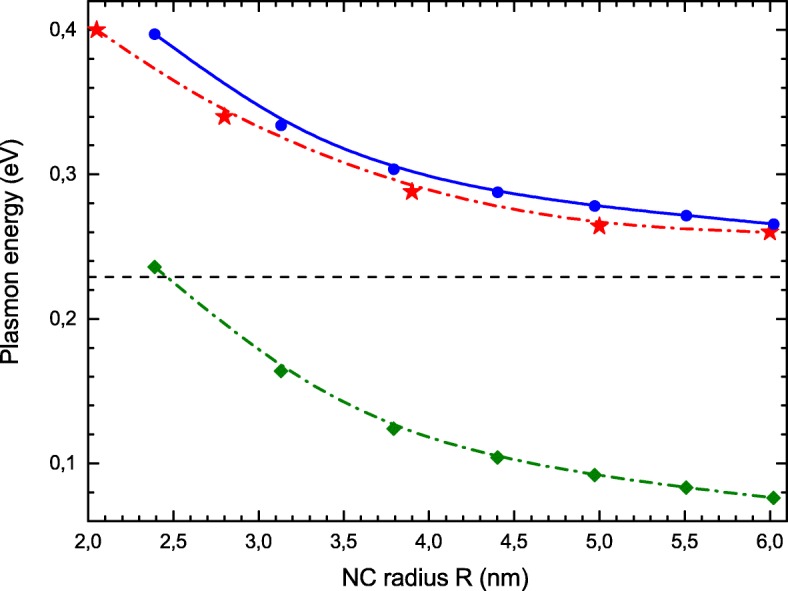
Size dependence of LSPR energy. Experimental values [21] (red stars), RPA with local exchange (blue circles), energies of single-particle transition (green diamonds in lower curve). Classical value (13) is shown by the horizontal dashed line

In Fig. 3, we compare the positions of the photoabsorption maxima, *ω*_*res*_, calculated here (blue circles) with experimental results of [21] (red stars). One observes a remarkable agreement between experimental data and our theoretical results. For comparison, we also plot here the energies *Δ* of single-particle transition (green squares). Electron correlations considerably increase the collective excitation energy comparatively to *Δ*. A simple analysis of RPA Eq. (7) explains this observation. If we consider only the main optical transition from *j*=(1,*l*_*max*_) to *j*=(1,*l*_*max*_+1) states, the RPA Eq. (7) reduces to a 2×2 matrix equation, which eigenvalue *ω* is simply:

11 $$ \omega^{2}=\Delta^{2} +2V\Delta,   $$

where $V=\left \langle ij\left | \hat {v}\right | ij\right \rangle $ denotes the RPA Coulomb matrix element. The first term in the r.h.s. of Eq. (11) gives the single-particle contribution whereas the second term results from particle-hole interactions. Their ratio can be estimated by taking the electron radial coordinate at the surface, *r*=*R* in the RPA matrix element *V*, and neglecting the exchange term. This estimation yields

12 $$ \frac{2V}{\Delta} \simeq \frac{4R\varepsilon_{i}}{a_{0}(\varepsilon_{i}+2\varepsilon_{m})} \simeq \frac{2R}{a_{0}}.   $$

Thus, the contribution of electron correlations dominates if *R*≫*a*_0_. In the limit of large NCs, the electron density distribution is concentrated at the NC surface; therefore, according to Eq. (12), the resonance energy in Eq. (11) tends to

13 $$ \omega = \sqrt{\frac{2\hbar^{2}e^{2}N}{m_{e}^{*}(\varepsilon_{i}+2\varepsilon_{m})R^{3}}},   $$

which exactly coincides with the classical dipole plasmon frequency in thin spherical shell [37]. This plasmon mode corresponds to a tangential electron vibration. This way, it is similar to the surface plasmon resonance in *C*_60_ molecules, where the resonance frequency is well described by the equation analogous to Eq. (13) [38]. Note that like the fullerene case [39], this plasmon mode gathers two thirds of the total oscillation strength (see Fig. 2). It happens because this oscillation involves only the angular degrees of freedom, keeping the radial motion unperturbed.

On the contrary to the well-known dipole surface plasmon mode in homogeneous spheres, which is purely translational, the mode of present consideration is compressional. The induced density produces an electric field parallel to the surface that plays the role of restoring force for this plasmon mode. Moreover, the purely quantum variation of local Fermi level due to induced density oscillations contributes to the restoring force. The corresponding quantum pressure contribution to the resonance frequency is given by the *Δ* term in Eq. (11). It is smaller than the Coulomb contribution in all considered NCs because of the smallness of the effective Bohr radius *a*_0_ in ZnO. However, in NCs with larger value of *a*_0_, one could observe the transition from classical dipole plasmon resonance to quantum confinement regime at *R*∼*a*_0_. It is worth noting that in the case of doped NCs, the parameter *a*_0_/*R* which controls the classical/quantum nature of the dipole resonance depends only on the NC size and does not depend on the number of free carriers *N*.

In Fig. 3, the horizontal line refers to the classical plasmon resonance energy (13). The blue shift of the resonance frequency against its classical value is caused by two quantum effects, the quantum pressure contribution discussed above and the decrease of average electron radius. The latter happens because electrons are pushed in the NC volume due to quantum reflection from the boundary, such that 〈*r*〉≃*R*−2*a*_0_. This effect increases the matrix element *V* which in turn increases the resonance frequency. Roughly, this effect can be reproduced by replacing the NC radius *R* in the denominator of Eq. (13) by 〈*r*〉. According to Eqs. (11)–(13), both effects provide the blue frequency shift proportional to the inverse NC radius ∝1/*R*. However, numerically, the contribution of the last one is the largest.

## Conclusions

To conclude this letter, we have worked out a theory that nicely predicts the strong dipole resonance observed in highly n-doped colloidal ZnO NCs. The new type of surface dipole plasmon excitations have been theoretically studied using many-body quantum approach. We have demonstrated that the strong Coulomb repulsion in photodoped ZnO nanocrystals leads to specific ground state electron distribution localized in a thin surface layer close to the inner surface. When the dipole is excited, this electronic distribution sustains a collective plasmon oscillation which is essentially formed of angular motion. Transition of this surface plasmon mode from the classical to quantum confinement regime is governed by a single parameter equal to the ratio of nanocrystal size to effective Bohr radius. Electron reflection from the NC interface reduces the electronic shell radius. Besides, the variation of the local Fermi level gives an additional contribution to the plasmon oscillator restoring force. These quantum effects lead to the size dependence of resonance plasmon frequency which is a remarkable agreement with experimental observations. In the limit of large NC radius, the resonance line tends smoothly to the classical plasmon frequency of a charged shell of infinitesimal width.

## Abbreviations

LDA: Local density approximation
LSPR: Localized surface plasmon resonance
NC: Nanocrystal
RPA: Random phase approximation
